# Cross-tissue and cross-species analysis of gene expression in skeletal muscle and electric organ of African weakly-electric fish (Teleostei; Mormyridae)

**DOI:** 10.1186/s12864-015-1858-9

**Published:** 2015-09-03

**Authors:** Francesco Lamanna, Frank Kirschbaum, Isabelle Waurick, Christoph Dieterich, Ralph Tiedemann

**Affiliations:** Unit of Evolutionary Biology/Systematic Zoology, Institute of Biochemistry and Biology, University of Potsdam, Karl-Liebknecht-Strasse 24-25, Potsdam, Germany; Department of Crop and Animal Sciences, Faculty of Horticulture and Agriculture, Humboldt University of Berlin, Philippstrasse 13, Berlin, Germany; Museum für Naturkunde, Invalidenstraße 43, Berlin, Germany; Max Delbrück Center for Molecular Medicine, Robert-Rössle-Strasse 10, Berlin-Buch, Germany; Max Planck Institute for Biology of Ageing, Joseph-Stelzmann-Strasse 9B, Cologne, Germany

## Abstract

**Background:**

African weakly-electric fishes of the family Mormyridae are able to produce and perceive weak electric signals (typically less than one volt in amplitude) owing to the presence of a specialized, muscle-derived electric organ (EO) in their tail region. Such electric signals, also known as Electric Organ Discharges (EODs), are used for objects/prey localization, for the identification of conspecifics, and in social and reproductive behaviour. This feature might have promoted the adaptive radiation of this family by acting as an effective pre-zygotic isolation mechanism. Despite the physiological and evolutionary importance of this trait, the investigation of the genetic basis of its function and modification has so far remained limited. In this study, we aim at: i) identifying constitutive differences in terms of gene expression between electric organ and skeletal muscle (SM) in two mormyrid species of the genus *Campylomormyrus*: *C. compressirostris* and *C. tshokwe*, and ii) exploring cross-specific patterns of gene expression within the two tissues among *C. compressirostris*, *C. tshokwe*, and the outgroup species *Gnathonemus petersii*.

**Results:**

Twelve paired-end (100 bp) strand-specific RNA-seq Illumina libraries were sequenced, producing circa 330 M quality-filtered short read pairs. The obtained reads were assembled *de novo* into four reference transcriptomes. *In silico* cross-tissue DE-analysis allowed us to identify 271 shared differentially expressed genes between EO and SM in *C. compressirostris* and *C.tshokwe.* Many of these genes correspond to myogenic factors, ion channels and pumps, and genes involved in several metabolic pathways. Cross-species analysis has revealed that the electric organ transcriptome is more variable in terms of gene expression levels across species than the skeletal muscle transcriptome.

**Conclusions:**

The data obtained indicate that: i) the loss of contractile activity and the decoupling of the excitation-contraction processes are reflected by the down-regulation of the corresponding genes in the electric organ’s transcriptome; ii) the metabolic activity of the EO might be specialized towards the production and turn-over of membrane structures; iii) several ion channels are highly expressed in the EO in order to increase excitability; iv) several myogenic factors might be down-regulated by transcription repressors in the EO.

**Electronic supplementary material:**

The online version of this article (doi:10.1186/s12864-015-1858-9) contains supplementary material, which is available to authorized users.

## Background

Bioelectrogenesis (i.e., the ability to produce strong or weak electric signals by specialized organs) has evolved several times independently in aquatic vertebrates [1]. In fact, it can be observed in the marine electric rays (Torpediniformes) and skates (Rajiformes), in the African freshwater Mormyridae and Gymnarchidae (Osteoglossiformes; Mormyroidea), in the South American knifefishes (Gymnotiformes), in several catfish species (Siluriformes), and in few marine stargazers (Perciformes; Uranoscopidae). In all the above-mentioned groups, electric organs originate from myogenic tissue; the only exception are members of the family Apteronotidae (Gymnotiformes), where the electric organs are formed by modified spinal motor neurons [2]. The amount of excitable cells within each electric organ determines the electric potential of an EOD, which can range from few millivolts to several hundreds of volts (e.g., in the electric eel *Electrophorus electricus*) [3].

African weakly-electric fishes of the family Mormyridae constitute a group of teleost fishes, formed by approximately 200 species [4], all endemic to African riverine and, partially, lacustrine systems. As their name suggests, they are able to produce only weak electric fields, in the order of millivolts to few volts, which are not used for predation or defence. The cells forming their electric organ are compressed disk-like cells commonly called electrocytes. In many species they are longitudinally stacked behind each other in order to form columns of cells embedded within tubes of isolating connective tissue. The synchronous activity of each electrocyte defines the output of the electric organ, known as Electric Organ Discharge (EOD) [5]. Such weak pulses are mainly employed for the localization and discrimination of objects in water (active electrolocation) [6], for the recognition of conspecific individuals [7, 8], and in social and reproductive behaviour [9, 10].

Besides these functional roles, the EODs of Mormyrids display remarkable levels of differentiation in terms of shape and length across different species [11]. Such differences, which can be dramatic even among closely related species, are considered to have acted as effective prezygotic isolation mechanisms, promoting thus the adaptive radiation observed in this family, facilitated by either ecological speciation [12–14] or speciation driven by sexual selection [15].

In all mormyrids, the adult electric organ is located in the caudal peduncle and is formed by four columns of electrocytes, two dorsal and two ventral ones. Each electrocyte is innervated by electromotoneurons originating in the spinal cord [3]. Electric organs arise in juvenile fishes from several myomeres of the deep lateral muscle; their myogenic origin is confirmed by the presence of disorganized myofibrils within the electrocytes [16, 17].

During the last decade, several studies have investigated the genetic basis of EOD function and evolution [18–22]. Some of them have stressed the importance of duplication in a class of sodium channel genes for the origin of EOD production and diversification in weakly-electric fishes, by revealing the presence of important functional substitutions across paralogs and by discovering their differential patterns of expression between electric organ (EO) and skeletal muscle (SM) [18, 19, 22]. More recent studies however [20, 21], based on high throughput genomic technologies (e.g., SSH; RNA-Seq) have identified many differentially expressed genes, belonging to multiple functional classes (e.g., transcription factors; ion channels; sarcomeric proteins), between skeletal muscle and electric organ in several weakly-electric species (including representatives of Mormyridae). All studies conducted so far have been focusing on gene expression differences between two tissues –i.e., skeletal muscle and electric organ- within a species (cross-tissue approach), overlooking possible differences in the same tissue across different species (cross-species approach). However, tissue-specific gene expression differences across several species might underlie important phenotypic differences [23] which, in the case of the electric organ of mormyrid fishes, could explain the species-specific variability of EODs.

**Fig. 1:**
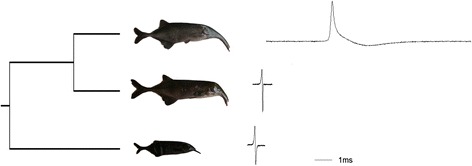
Analyzed species. The three species analysed in this study, with their relative EODs. From bottom to top: *G. petersii*, *C. compressirostris*, *C. tshokwe*

The aim of the present work is twofold; first we aim at exploring the differential patterns of gene expression between skeletal muscle and electric organ (cross-tissue comparison) in adult specimens belonging to the mormyrid genus *Campylomormyrus* (*C. compressirostris* and *C. tshokwe*). We focus then, on the identification of the differentially expressed genes, that are in common between the two species, and that might be responsible for the functional differences between the two tissues, and compare them to the results obtained by previous studies. The second task is to find differences in gene expression among three mormyrid species (*C.compressirostris*, *C.tshokwe*, and the outgroup species *Gnathonemus petersii*; Fig. 1) for the skeletal muscle and electric organ separately (cross-species comparison), and identify genes potentially related to phenotypic differences in EOD shape and duration.

## Results

### Transcriptome sequencing and assembly

**Fig. 2:**
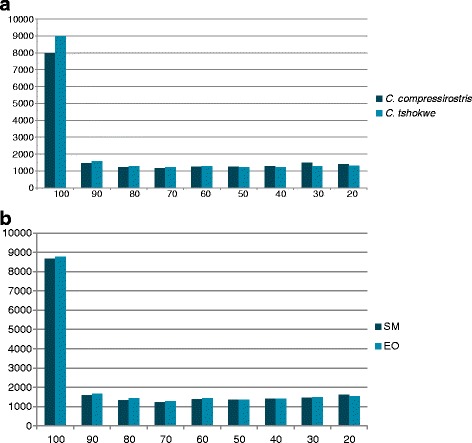
Distribution of length coverage between *Campylomormyrus* Trinity transcripts and corresponding top-blast hits (*D. rerio* proteome). Histogram showing the distribution of the percent in length of the sequences in the *D. rerio* proteome that aligns to the assembled Trinity contigs. Numbers on the x-axis indicate the upper limit of the binned interval (e.g., 100 is the upper value of the interval 100–91). **a** Cross-tissue comparison. **b** Cross-species comparison

Sequencing of the twelve cDNA libraries produced a total amount of 371,043,357raw read pairs, resulting in 330,595,546 quality-filtered read pairs (89.1 %); see Additional file 1 for per library sequencing statistics. Trinity assembly resulted in 260,598 and 369,030 contigs for *C. compressirostris* and *C.tshokwe* cross-tissue transcriptomes respectively (Table 1); 357,832 and 399,878 contigs were obtained for the SM and EO cross-species assemblies respectively (Table 2). Contigs were then compared to the *Danio rerio* proteome, retrieving 18,458 and 19,363 unique proteins for *C. compressirostris* and *C.tshokwe* respectively; of these retrieved matches, 7971 (43.1 %) and 8993 (46.4 %) hits corresponded to full or nearly full-length coding sequences (Fig. 2a). For the cross-species assemblies, 20,023 and 20,352 contigs, for the SM and EO respectively, matched unique proteins in the *D. rerio* proteome, with 8662 (43.3 %) and 8768 (43.1 %) hits corresponding to full or nearly full-length coding sequences (Fig. 2b).

**Table 1:** Assembly statistics for the cross-tissue comparison

	*C. compressirostris*	*C. tshokwe*
Trinity contigs	260,598	369,030
# of retrieved ORFs	139,963	228,306
# of unique PFAM domains	9,986	9,941
# of contigs matching *D. rerio* proteome	108,705	159,263
# of unique hits to *D. rerio* proteome	18,458	19,363
N50	3,010	3,597
Average contig length	1,392.16	1,672.28
Total assembled bases	362,794,286	617,123,151

**Table 2:** Assembly statistics for the cross-species comparison

	SM	EO
Trinity contigs	357,832	399,878
# of retrieved ORFs	150,068	182,682
# of unique PFAM domains	10,374	10,752
# of contigs matching *D. rerio* proteome	193,516	232,755
# of unique hits to *D. rerio* proteome	20,023	20,352
N50	2,299	2,345
Average contig length	1,119.38	1,149.25
Total assembled bases	400,549,148	459,560,613

**Table 3:** Electrical activity

Gene	Protein name	Expression in EO	Pathway/Function	Disrupted phenotype	Reference
atp1a2a	ATPase, Na+/K+ transporting, alpha 2a polypeptide	+	Ion channel transport	Impaired depolarization of the resting membrane potential in slow-twitch fibers of skeletal muscles.	[50]
chrna7	cholinergic receptor, nicotinic, alpha 7 (neuronal)	+	Activation of Nicotinic Acetylcholine Receptors		
kcnj9	potassium inwardly-rectifying channel, subfamily J, member 9	+	Potassium Channels; GABA receptor activation		
kcnq5a	potassium voltage-gated channel, KQT-like subfamily, member 5a	+	Potassium Channels; Synaptic transmission ion currents		
grik3	Glutamate Receptor, Ionotropic, Kainate 3	+	Transmission across Chemical Synapses		
scn4aa	sodium channel, voltage-gated, type IV, alpha, a	+	Ion channel transport; Axon guidance		
kcna3	potassium voltage-gated channel, shaker-related subfamily, member 3	-	Potassium Channels; Transmission across Chemical Synapses		
kcnj12	potassium inwardly-rectifying channel, subfamily J, member 12	-	Potassium Channels; GABA receptor activation		
cacna2d2	calcium channel, voltage-dependent, alpha 2/delta subunit 2	-	Ion channel transport		

For each of the shared differentially expressed gene are reported: the gene and protein names obtained from the top hit blast results against the proteome of *D. rerio*; whether it is up(+)- or down(−)- regulated in the EO; its function or pathway (or both when available); the phenotypic effect on *D. rerio* of its mis-expression (when available)

### Differential Expression (DE) analysis

#### Cross-tissue comparison

**Fig. 3:**
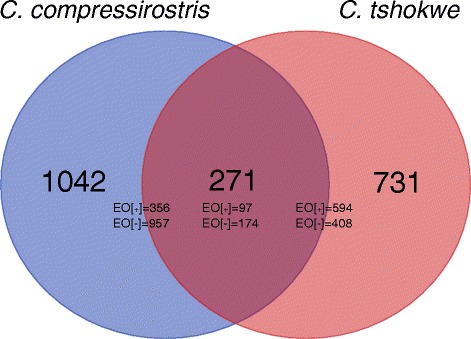
Number of differentially expressed genes (Cross-tissue). Venn diagram showing the amount of differentially expressed genes within each *Campylomormyrus* species’ transcriptome (full circles) and the amount of differentially expressed genes shared between the two *Campylomormyrus* species (overlapping area). The amount of genes that are up (EO[+])- or down (EO[−])-regulated in the electric organ are reported for each dataset

After transcript quantification with RSEM and DE-analysis with edgeR, 1313 transcripts resulted to be differentially expressed between EO and SM in *C. compressirostris* (356 up-regulated in EO and 957 down-regulated in EO) and 1002 in *C. tshokwe* (594 up-regulated in EO and 408 down-regulated in EO). Of all differentially expressed transcripts, 271 resulted to be shared between the two species (97 up-regulated in EO and 174 down-regulated in EO) (Fig. 3).

#### Cross-species comparison

**Fig. 4:**
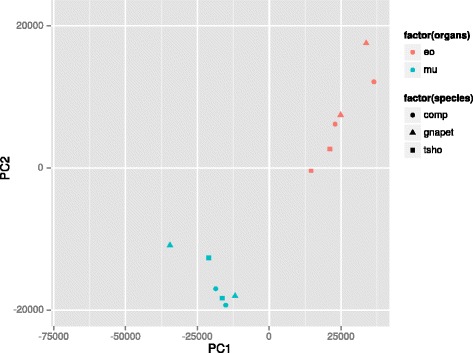
Principal component analysis of expression levels. Gnapet = *G. petersii*; comp = *C. compressirostris*; tsho = *C. tshokwe*; eo = electric organ; mu = skeletal muscle

**Fig. 5:**
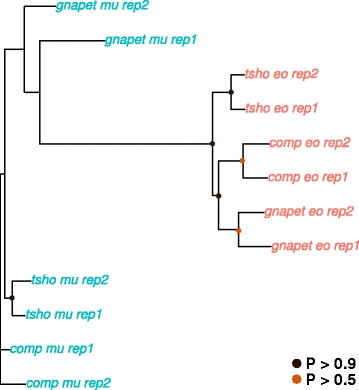
Neighbour-joining analysis of expression levels. Neighbour-joining tree based on pairwise distance matrix (1 – ρ, Spearman’s correlation coefficient) for EO and SM expression values. Bootstrap replicates = 10,000. Circles at nodes indicate bootstrap support. Gnapet = *G. petersii*; comp = *C. compressirostris*; tsho = *C. tshokwe*; eo = electric organ; mu = skeletal muscle; rep = replicate

In order to obtain an initial overview of transcriptome-wide gene expression patterns, we performed a principal component analysis on the expression levels of all assessed transcripts with non-zero levels in both assemblies (see methods for details). The results clearly separate the data according to tissue rather than species (Fig. 4). A distance matrix was then obtained from the same dataset and a neighbour-joining gene expression tree was built (see methods) in order to analyse global evolutionary trends in more detail. The obtained tree (Fig. 5) shows a clustering pattern for the EO data where species characterized by similar EODs (*G. petersii*, *C. compressirostris*) are grouped together, whereas the species with a rather different EOD (*C. tshokwe*) forms an isolated cluster. SM data, on the other hand, do not seem to form any particular clustering scheme.

**Fig. 6:**
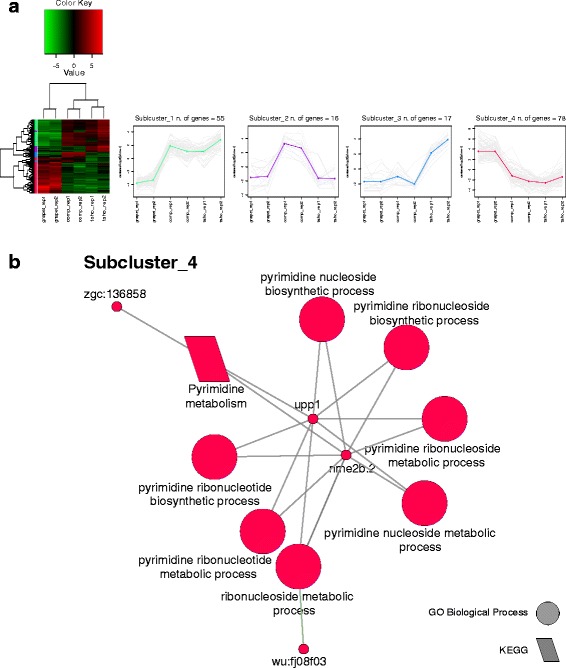
Cross-species analysis (SM). **a** Results of the DE analysis. Left: heatmap showing the differentially expressed genes clustered by expression levels. Expression sub-clusters obtained from k-mean clustering. Each cluster groups together genes characterized by similar expression levels. **b** Network showing significantly enriched terms and their relative genes for each sub-cluster

**Fig. 7:**
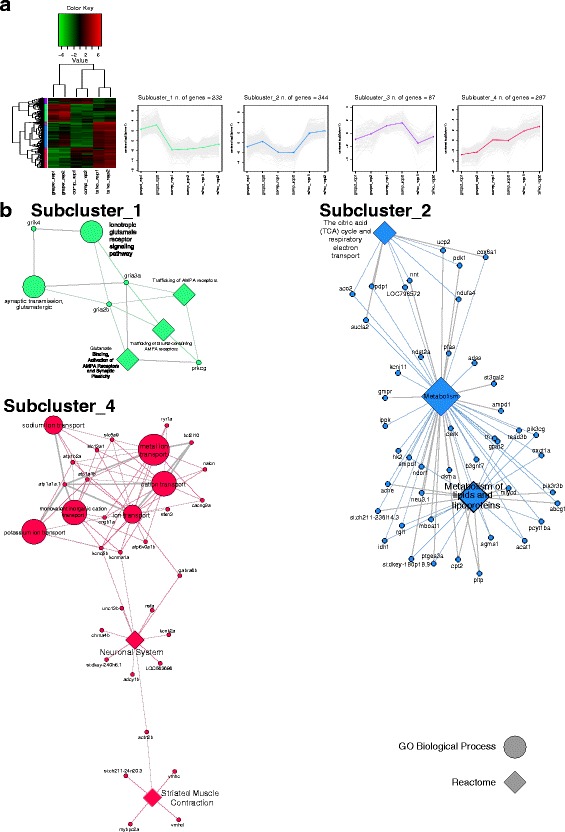
Cross-species analysis (EO). **a** Results of the DE analysis. Left: heatmap showing the differentially expressed genes clustered by expression levels. Expression sub-clusters obtained from k-mean clustering. Each cluster groups together genes characterized by similar expression levels. **b** Networks showing significantly enriched terms and their relative genes for each sub-cluster

After DE analysis, 166 and 950 genes resulted to be differentially expressed across the three analysed species for SM and EO, respectively. Within the skeletal muscle 78 genes are up-regulated in *G. petersii*, 16 in *C. compressirostris*, 17 in *C. tshokwe*, and 55 in *C. compressirostris* and *C. tshokwe* together (Fig. 6a). As far as the electric organ is concerned, 232 genes are up-regulated in *G. petersii*, 87 in *C. compressirostris*, and 631 in *C. tshokwe* (Fig. 7a).

### Functional annotation

#### Cross-tissue comparison

**Fig. 8:**
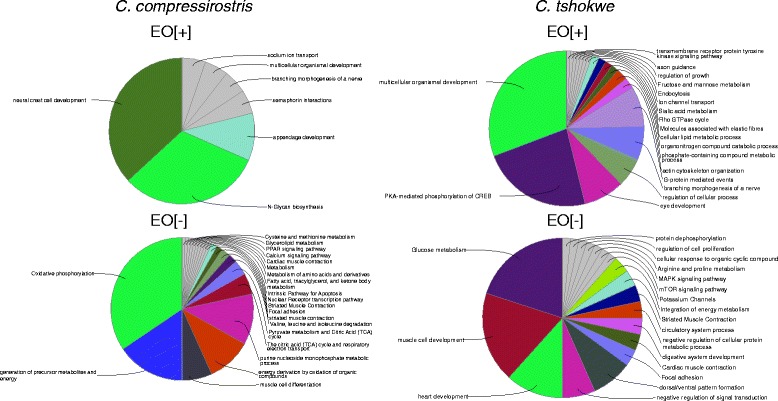
Functional annotation results (Cross-tissue). Pie charts showing the composition in terms of enriched functional categories (GO) and pathways (KEGG, Reactome) for each cluster of differentially expressed genes. EO[+] = up-regulated in the electric organ; EO [−] = down-regulated in the electric organ

In order to identify over-represented functional categories and pathways within the *C. compressirostris* and *C. tshokwe* transcriptomes, the sets of up- and down-regulated genes in the electric organ were subject to an enrichment analysis involving Gene Ontology categories, as well as Reactome and KEGG biological pathways (Fig. 8). The number of retrieved categories/pathways is proportional to the amount of differentially expressed genes present in each analysed set; with fewer categories for the EO up-regulated genes compared to the EO down-regulated genes in *C. compressirostris,* and a similar number of categories identified between the two sets in *C. tshokwe*. For both species the most represented categories within the EO up-regulated genes are related to ion channel transport (e.g., sodium ion transport), multicellular organismal development, development and patterning of nerves (branching morphogenesis of a nerve; semaphorin interactions; axon guidance). The genes found to be down-regulated in the EO regard mainly functional classes like: metabolic pathways specific to muscle tissue (oxidative phosphorylation, pyruvate metabolism, calcium signalling pathway), and muscle specific categories (muscle cell differentiation, striated muscle contraction, cardiac muscle contraction).

Given the information provided by the category-based functional annotation, and to better understand the functional differences in terms of gene expression between EO and SM, independently from the species analysed, a literature search was performed on the shared set of differentially expressed genes between *C. compressirostris* and *C. tshokwe.* For each gene, phenotypic information consequent to its mis-expression (e.g., via knockdowns or non-sense mutations) in *D. rerio* was retrieved from the “Zebrafish Model Organism Database” (ZFIN; http://zfin.org/). All shared genes were divided into five “general” functional classes, which synthesize the categories reported in Fig. 8. The chosen categories are: “electrical activity” (genes responsible for the differential accumulation and transfer of ions across the plasma membrane), “muscular activity” (genes important for keeping a functional muscle phenotype), “metabolism” (genes involved in metabolic pathways), “transcription factors” (genes regulating gene expression) and “signal transduction” (molecules involved in signalling pathways) (Tables 2, 3, 4, 5 and 6). Many of the genes present in the category “electrical activity” are up-regulated in the EO (Table 2), they include genes coding for Na+/K+ pumps (*atp1a2a*), voltage-gated sodium (*scn4aa*) and potassium channels (*kcnq5a*) and cholinergic receptors (*chrna7*). However, other voltage-gated ion channels result to be down-regulated in the EO (*kcna3*, *cacna2d2*). There are then two members of the subfamily J of inwardly-rectifying potassium channels that show distinct patterns of expression, with one member (*kcnj9*) up-regulated in EO and the other (*kcnj12*) down-regulated. All the genes included in the class “muscular activity” are down-regulated in the EO (Table 3). As far as the “metabolism” genes are concerned (Table 4), most of the EO up-regulated transcripts are involved in the metabolism of fatty acids, glycerol, and phospholipids (e.g., *acsl3b*, *gdpd4a*, *cds1*), whereas the down-regulated transcripts are more involved in muscle-specific, energy production processes, like glycolysis (*aldoab*) and gluconeogenesis (*gpib*). Among transcription factors (Table 5), two of the four known myogenic factors (transcription factors that activate the expression of sarcomeric proteins), are down-regulated in the EO (*myog*, *myf6*), while the other two (*myoD, myf5*) do not show significant differences in expression between the two tissues. Two basic helix-loop-helix (bHLH) transcription factors (*hey1*, *hes6*) and one co-factor (*her6*) are up-regulated in the electric organ. Two myocyte enhancer factors (*mef2aa*, *mef2b*) show high levels of expression in the EO, whereas two regulators of SM cell proliferation (*six1b*, *six4b*) are lowly expressed in the EO. Most of the EO up-regulated genes involved in signal transduction (Additional file 9) belong to the G-protein coupled receptor (GPCR) signalling pathway (e.g., *arhgef7a*, *arhgef7b*, *gpr22*) and to the fibroblast growth-factor receptor (FGFR) signalling pathway (e.g., *fgf8a*, *kal1b*)

**Table 4:** Muscular activity

Gene	Protein name	Expression in EO	Pathway/Function	Disrupted phenotype	Reference
atp2a1	ATPase, Ca++ transporting, cardiac muscle, fast twitch 1	-	Muscle contraction	Abnormal locomotion	[51]
atp2a2	ATPase, Ca++ transporting, cardiac muscle, slow twitch 2a	-	regulation of heart contraction	Abnormal heart development	[31]
casq1a	calsequestrin 1a	-	Calcium homeostasis		
jph1a	junctophilin 1a	-	structural constituent of muscle		
jph1b	junctophilin 1b	-	structural constituent of muscle		
myl2a	myosin, light chain 2a, regulatory, cardiac, slow	-	Striated Muscle Contraction		
mybpc2a	myosin binding protein C, fast type a	-	Striated Muscle Contraction		
mybpc3	myosin binding protein C, cardiac	-	Cardiac muscle contraction	Abnormal heart development	[52]
myhb	myosin, heavy chain b	-	Striated Muscle Contraction		
myl10	myosin, light chain 10, regulatory	-	Regulation of actin cytoskeleton; Focal adhesion		
myl12.2	myosin, light chain 12, genome duplicate 2	-	Striated Muscle Contraction		
mylk2	myosin light chain kinase 2	-	Focal adhesion; Regulation of actin cytoskeleton		
mylk3	myosin light chain kinase 3	-	Focal adhesion; Regulation of actin cytoskeleton	Cardiac sarcomere disruption	[53]
mylpfb	myosin light chain, phosphorylatable, fast skeletal muscle b	-	Focal adhesion; Regulation of actin cytoskeleton		
myo18ab	myosin XVIIIAb	-	Signaling by FGFR		
myoz3a	myozenin 3a	-	Calcineurin signaling		
nexn	nexilin (F actin binding protein)	-	cardiac muscle fiber development		
parvb	parvin, beta	-	Focal adhesion; Cell junction organization	Abnormal trunk musculature development	[54]
pdlim3b	PDZ and LIM domain 3b	-			
pdlim5b	PDZ and LIM domain 5b	-			
pvalb3	parvalbumin 3	-	calcium ion homeostasis		
ryr1a	ryanodine receptor 1a (skeletal)	-	calcium ion channel transport	Abnormal trunk musculature development	[32]
ryr1b	ryanodine receptor 1b (skeletal)	-	calcium ion channel transport	Abnormal trunk musculature development	[33]
tnnc2	troponin C type 2 (fast)	-	Striated Muscle Contraction		
smpx	small muscle protein, X-linked	-	Striated Muscle Contraction		
smyd1b	SET and MYND domain containing 1b	-	Muscle Development	Thick myosin filament disorganization	[55]
srl	sarcalumenin	-	calcium ion homeostasis		
stac3	SH3 and cysteine rich domain 3	-	Striated Muscle Contraction	Excitation–contraction coupling disruption	[34]
tcap	titin-cap (telethonin)	-	Striated Muscle Contraction	Myofibril disorganization	[30]
tmod4	tropomodulin 4 (muscle)	-	Muscle contraction		
tnnc1b	troponin C type 1b (slow)	-	Muscle contraction		
tnni2b.2	troponin I type 2b (skeletal, fast), tandem duplicate 2	-	Striated Muscle Contraction		
tnnt1	troponin T type 1 (skeletal, slow)	-	Muscle contraction		
tnnt3b	troponin T type 3b (skeletal, fast)	-	Striated Muscle Contraction		
tpm1	tropomyosin 1 (alpha)	-	Striated Muscle Contraction		
tpm2	tropomyosin 2 (beta)	-	Striated Muscle Contraction		
trdn	triadin	-	Muscle contraction		
trim54	tripartite motif containing 54	-	Titin-kinase regulation		
xirp1	xin actin-binding repeat containing 1	-			
myl13	myosin, light chain 13	-			

For each of the shared differentially expressed gene are reported: the gene and protein names obtained from the top hit blast results against the proteome of *D. rerio*; whether it is up(+)- or down(−)- regulated in the EO; its function or pathway (or both when available); the phenotypic effect on *D. rerio* of its mis-expression (when available)

**Table 5:** Metabolism

Gene	Protein name	Expression in EO	Pathway/Function	Disrupted phenotype	Reference
acsbg2	acyl-CoA synthetase bubblegum family member 2	-	Fatty acid metabolism		
acsl3b	acyl-CoA synthetase long-chain family member 3b	+	Fatty acid metabolism		
acy1	Aminoacylase-1	-	Aminoacids metabolism		
adssl1	adenylosuccinate synthase like 1	-	Purine metabolism		
aldoab	Fructose-bisphosphate aldolase	-	Glycolysis		
ampd1	Adenosine monophosphate deaminase 1 (Isoform M)	-	Purine metabolism		
aoc2	amine oxidase, copper containing 2	+	beta-Alanine metabolism		
cds1	CDP-Diacylglycerol Synthase 1	+	Glycerophospholipid biosynthesis	Imperfect angiogenesis	[56]
ckma	creatine kinase, muscle a	-	Metabolism of amino acids and derivatives		
ckmt2a	creatine kinase, mitochondrial 2a (sarcomeric)	-	Metabolism of amino acids and derivatives		
cox4i2	cytochrome c oxidase subunit IV isoform 2	-	Oxidative phosphorylation		
cpt2	carnitine palmitoyltransferase 2	+	Fatty acid beta-oxidation		
cyp24a1	cytochrome P450, family 24, subfamily A, polypeptide 1	-	Steroid biosynthesis		
dhrs9	dehydrogenase/reductase (SDR family) member 9	+	Retinol metabolism		
gdpd4a	glycerophosphodiester phosphodiesterase domain containing 4a	+	Glycerol metabolism		
gdpd5a	glycerophosphodiester phosphodiesterase domain containing 5a	+	Glycerol metabolism		
gdpd5b	glycerophosphodiester phosphodiesterase domain containing 5b	+	Glycerol metabolism		
glo1	Glyoxalase 1	-	Pyruvate metabolism		
glud1b	glutamate dehydrogenase 1b	-	Nitrogen metabolism		
got2a	glutamic-oxaloacetic transaminase 2a, mitochondrial	-	Glucose metabolism; aminoacids metabolism		
gpib	glucose-6-phosphate isomerase b	-	Gluconeogenesis		
idi1	isopentenyl-diphosphate delta isomerase 1	-	Cholesterol biosynthesis		
man1a1	mannosidase, alpha, class 1A, member 1	+	N-Glycan biosynthesis		
me3	malic enzyme 1, NADP(+)-dependent, cytosolic	-	Pyruvate metabolism		
pcyox1	prenylcysteine oxidase 1	-	Terpenoid backbone biosynthesis		
pfkfb1	6-phosphofructo-2-kinase/fructose-2,6-biphosphatase 1	-	Glycolysis		
pgam2	phosphoglycerate mutase 2 (muscle)	-	Glycolysis and Gluconeogenesis		
pgm5	Phosphoglucomutase 5	-	Glucuronidation	Failure in myofibril assembly	[57]
ucp3	uncoupling protein 3	-	Respiratory electron transport		
ugp2b	UDP-glucose pyrophosphorylase 2b	-	Glucose metabolism		
gyg1a	glycogenin 1a	-	Glycogen Metabolism		
mid1ip1l	MID1 interacting protein 1, like	-	lipid metabolic process		

For each of the shared differentially expressed gene are reported: the gene and protein names obtained from the top hit blast results against the proteome of *D. rerio*; whether it is up(+)- or down(−)- regulated in the EO; its function or pathway (or both when available); the phenotypic effect on *D. rerio* of its mis-expression (when available)

**Table 6:** Transcription factors

Gene	Protein name	Expression in EO	Pathway/Function	Disrupted phenotype	Reference
eng1b	engrailed homeobox 1b	+	neuron fate commitment		
her6	hairy-related 6	+	Notch signaling pathway		
hes6	hes family bHLH transcription factor 6	+	Notch signaling pathway		
hey1	hes-related family bHLH transcription factor with YRPW motif 1	+	Notch signaling pathway		
hipk2	homeodomain interacting protein kinase 2	+	Wnt signaling pathway; p53 Signaling; ERK Signaling	Induced apoptosis	[58]
hoxc11a	homeobox C11a	+			
hoxd11a	homeobox D11a	+			
mef2aa	myocyte enhancer factor 2aa	+	Signaling by FGFR	Abnormal development of posterior somites	[29]
mef2b	myocyte enhancer factor 2b	+	miRs in Muscle Cell Differentiation		
rb1	retinoblastoma 1	+	E2F mediated regulation of DNA replication; Cell cycle	Abnormal retina development	[59]
taf6	TAF6 RNA polymerase II, TATA box binding protein (TBP)-associated factor	+	GPCR Pathway		
arxa	aristaless related homeobox a	-	Axon guidance	Abnormal dopaminergic neurons development	[60]
klf15	Kruppel-like factor 15	-	Adipogenesis		
pbxip1	pre-B-cell leukemia homeobox interacting protein 1	-			
myf6	myogenic factor 6	-	Myogenesis	Disrupted myogenesis	[28]
myog	myogenin	-	Myogenesis	Disrupted myogenesis	[27]
nfatc1	nuclear factor of activated T-cells, cytoplasmic, calcineurin-dependent 1	-	Wnt signaling pathway		
nr0b2a	nuclear receptor subfamily 0, group B, member 2a	-	Nuclear Receptor transcription pathway; NOD-like Receptor Signaling Pathways		
pitx2	paired-like homeodomain 2	-	retinoic acid receptor signaling pathway	Abnormal eye and craniofacial development	[61]
rxrgb	retinoid X receptor, gamma b	-	steroid hormone receptor activity; retinoic acid receptor signaling pathway		
six1b	SIX homeobox 1b	-	regulation of skeletal muscle cell proliferation	Abnormal trunk musculature development	[62]
six4b	SIX homeobox 4b	-	regulation of skeletal muscle cell proliferation		
tbx15	T-box 15	-	regulation of transcription, DNA-templated		

For each of the shared differentially expressed gene are reported: the gene and protein names obtained from the top hit blast results against the proteome of *D. rerio*; whether it is up(+)- or down(−)- regulated in the EO; its function or pathway (or both when available); the phenotypic effect on *D. rerio* of its mis-expression (when available)

#### Cross-species comparison

The two sets of differentially expressed genes identified for SM and EO across the three analysed species were each partitioned into four sub-clusters with related expression patterns (see the methods section for details); each sub-cluster was then subjected to an enrichment analysis like the one described in the previous paragraph and in the methods section. Of the analysed sub-clusters for the SM dataset, one out of four showed significantly enriched terms, all related to nucleotides metabolic processes (Fig. 6b). Conversely, three out of four sub-clusters were significantly enriched in functional categories for the EO dataset (Fig. 7b). The most representative enriched functional categories are: glutamate receptor activity (sub-cluster 1); TCA cycle and fatty acid metabolism (sub-cluster 2); ion transport, neuronal system, and striated muscle contraction (sub-cluster 4).

For each of the analysed sub-clusters genes with known phenotypic effect in *D. rerio* or *H. sapiens* are reported in Table 7.

**Table 7:** Cross-species differentially expressed genes with known phenotypic effect

Gene	Protein	Tissue	Sub-cluster	Phenotype	Source
nme2b.2	NME/NM23 nucleoside diphosphate kinase 2b, tandem duplicate 2	SM	4	GTP biosynthesis	ZFIN
prkcg	protein kinase C, gamma	EO	1	AMPA-R kinetics	ZFIN
aco2	aconitase 2, mitochondrial	EO	2	Infantile cerebellar-retinal degeneration	OMIM
pdp1	pyruvate dehyrogenase phosphatase catalytic subunit 1	EO	2	Pyruvate dehydrogenase phosphatase deficiency	OMIM
nnt	nicotinamide nucleotide transhydrogenase	EO	2	Glucocorticoid deficiency 4	OMIM
sucla2	succinate-CoA ligase, ADP-forming, beta subunit	EO	2	Mitochondrial DNA depletion syndrome 5	OMIM
kcnj11	potassium inwardly-rectifying channel, subfamily J, member 11	EO	2	Diabetes mellitus	OMIM
ippk	inositol 1,3,4,5,6-pentakisphosphate 2-kinase	EO	2	Craniofacial development	ZFIN
smpd1	sphingomyelin phosphodiesterase 1, acid lysosomal	EO	2	Niemann-Pick disease	OMIM
th	tyrosine hydroxylase	EO	2	Adult brain function	ZFIN
ache	acetylcholinesterase	EO	2	Locomotion	ZFIN
oxct1a	3-oxoacid CoA transferase 1a	EO	2	Succinyl CoA:3-oxoacid CoA transferase deficiency	OMIM
mlycd	malonyl-CoA decarboxylase	EO	2	Malonyl-CoA decarboxylase deficiency	OMIM
idh1	isocitrate dehydrogenase 1 (NADP+), soluble	EO	2	Susceptibility to glioma	OMIM
cpt2	carnitine palmitoyltransferase 2	EO	2	Myopathy/Encephalopathy	OMIM
pltp	phospholipid transfer protein	EO	2	HDL cholesterol level	OMIM
acat1	acetyl-CoA acetyltransferase 1	EO	2	Alpha-methylacetoacetic aciduria	OMIM
atp1a1a.1	ATPase, Na+/K+ transporting, alpha 1a polypeptide, tandem duplicate 1	EO	3	Brain development	ZFIN
ryr1a	ryanodine receptor 1a (skeletal)	EO	3	Myopathy	ZFIN
bcl2l10	BCL2-like 10 (apoptosis facilitator)	EO	3	Cytoskeletal activity	ZFIN
nalcn	sodium leak channel, non-selective	EO	3	Hypotonia	OMIM
cngb1a	cyclic nucleotide gated channel beta 1a	EO	3	Retinitis pigmentosa 45	OMIM
kcnma1a	potassium large conductance calcium-activated channel, subfamily M, alpha member 1a	EO	3	Hearing sensitivity	ZFIN
kcnq5b	potassium voltage-gated channel, KQT-like subfamily, member 5b	EO	3	Cell membrane excitability	ZFIN
nsfa	N-ethylmaleimide-sensitive factor a	EO	3	Axon development	ZFIN
chrna4b	cholinergic receptor, nicotinic, alpha 4b	EO	3	Epilepsy	OMIM
adcy1b	adenylate cyclase 1b	EO	3	Deafness	OMIM
actn2b	actinin, alpha 2b	EO	3	Cardiomyopathy	OMIM
vmhc	ventricular myosin heavy chain	EO	3	Cardiomyopathy	OMIM

List of cross-species differentially expressed genes belonging to the terms obtained from the enrichment analysis. For each gene, we report: the analysed tissue; the relative sub-cluster as reported in Figs. 6 or 7; and the phenotypic effect of gene function disruption on *D. rerio* (ZFIN) or *Homo sapiens* (OMIM)

## Discussion

### Cross-tissue comparison

Functional annotation of the 271 differentially expressed genes that are shared between *C. compressirostris* and *C. tshokwe* has revealed marked differences within several functional categories, which are probably critical in determining the observed phenotypic differences between the electric organ and the skeletal muscle. Below, the functional implications of the differentially expressed genes, in the light of what is known from other fish models, are discussed.

#### Electrical activity

The up-regulation of the *atp1a2a* gene is explained by the fact that its product, the Na^+^/K^+^ ATP-ase, is fundamental for keeping the electrochemical gradient across the plasma membrane. Over-expression of this gene was already observed in the mormyrid fish *Brienomyrus brachyistius* [20], as well as in several species of south-American weakly-electric fishes (Gymnotiformes) [21]. Voltage-gated ion channels, on the other hand, are important for dissipating the electric potential generated by the ATP-ases and therefore for producing an EOD in response to an action potential. In the electric organ of the analyzed species, one gene coding for a voltage-gated sodium channel (*scn4aa*) is highly expressed in the electric organ; differential expression of this gene and of its paralog (*scn4ab*) between EO and SM was demonstrated by Zakon et al. [18] for mormyrid and gymnotiform fishes, and suggest the role of gene duplication followed by neo-functionalization as a main driver for the evolution of electric communication [19]. Other over-expressed genes that increase cell excitability are the potassium channels *kcnq5a* and *kcnj9*. The latter belongs to the family of inwardly rectifying potassium channels, a class of ion channels that favour the influx of K^+^ ions in the cell; up-regulation of members of this family was observed in the EO of the Electric eel (*E. electricus*) [24]*.*

#### Repression of muscular phenotype in the EO

Many of the differentially expressed transcription factors retrieved in this study are fundamental for the regulation of myogenic development. In particular, we have found that two bHLH transcription factors: *hey1* and *hes6*, in co-operation with *her6*, are up-regulated in the EO; these factors are known to negatively regulate the expression of myogenic factors in several model organisms [25, 26], including electric fish [21]. Two of the four known myogenic regulatory factors (MRFs: *myog*, *myf6*) are down-regulated in the EO, both genes are fundamental for muscle development and differentiation [27]; in particular, knock-down experiments on *myf6* have shown the degradation of posterior somites in *D. rerio* [28], the region where the adult EO originates [17]. Another gene important for muscle development is the myocyte enhancer factor *mef2aa*. Unlike MRFs, this gene is up-regulated in the electric organ of the two species analysed here, as well as in other electric fish species [20, 21], and it is also important for the correct development of posterior somites in *D. rerio* [29]. The concerted activity of transcriptional repressors and co-repressors of the myogenic program results in the down-regulation of genes coding for muscle specific proteins (Table 4), which finally determine the non-muscle characteristics of the EO like: i) the presence of few, non-contractile, myofibrils [17] (e.g., *tcap* [30]); ii) loss of calcium compartmentalization activity (e.g., *atp2a1*, *atp2a2*, *casq1a* [31]); and iii) decoupling of the excitation-contraction process (e.g., *ryr1*, *stac3*, *jph1* [32–34]).

#### Metabolic activity

The observed differences in terms of gene expression between EO and SM suggest that the metabolic machinery of the electric organ could be mainly devoted to the production and turn-over of membrane structures. Indeed, many of the metabolism-related genes up-regulated in the EO are involved in the metabolism of fatty acids (*acsbg2*, *acsl3b*, *cpt2*), glycerophospholipids (*cds1*, *gdpd4a*, *gdpd5a*, *gdpd5b*), and cholesterol (*idi1*). On the other hand, most of the SM up-regulated genes are involved in typical processes of muscle metabolism like: glycolysis (*aldoab*, *glo1*, *me3*); gluconeogenesis (*gpib*, *pgam2*); and aminoacids metabolism (*acy1*, *ckma*, *ckmt2a*).

### Cross-species comparison

The grouping pattern emerging from the principal component analysis, where expression levels tend to group in an “organ-wise” rather than a “species-wise” fashion, is putatively due to the fact that expression levels are conserved for the same organ across different species for functional reasons. A similar pattern was already observed for more tissues across broader phylogenetic distances [23].

The clustering scheme obtained from the neighbour-joining analysis for the EO data might be indicative of the observed differences in terms of EOD among the three species, which may be reflected in the expression levels of a conspicuous part of the EO transcriptome. Previous studies [23, 35] have revealed that, for most tissues, gene expression levels tend to accumulate over evolutionary time, such that more closely related species have more similar expression levels. However, for tissues characterized by increased levels of adaptation (e.g., testis and liver in mammals), expression trees tend to group according to phenotypic similarity [23].

The results of the enrichment analysis conducted on the expression clusters for the EO have revealed interesting results. In particular, sub-cluster 2 and sub-cluster 4 (Fig. 7b) are enriched in terms which might underlie the observed EOD differences across the three species; both sub-clusters are characterized by genes which are mainly up-regulated in the EO of *C. tshokwe*. The terms relative to sub-cluster 2 are all related to metabolic pathways, the metabolism of fatty acids in particular. Many of the observed genes are involved in the production and turnover of cell membranes (e.g., *smpd1*, *oxct1a*, *mlycd*, *cpt2*). Sub-cluster 4 is mainly characterized by genes involved in ion transport and neuronal function; of particular importance here are sodium/potassium ATPases (*atp1a1a*, *atp1a1b*, *atp1b2a*), as their over-expression in the EO of *C. tshokwe* might explain the higher amplitude observed in its EOD [36]. Other genes which may potentially influence EOD features are potassium channels (*kcnq5b*, *kcnma1a*, *kcnk2a*, *kcnj11*).

## Conclusions

The cross-tissue analysis of differentially expressed genes between skeletal muscle and electric organ in two species of African weakly-fishes suggests that: i) the loss of contractile activity and the decoupling of the excitation-contraction processes are reflected by the down-regulation of the corresponding genes in the electric organ; ii) the metabolic activity of the EO might be specialized towards the production and turn-over of membrane structures; iii) several ion channels are highly expressed in the EO in order to increase excitability; iv) several myogenic factors might be down-regulated by transcription repressors in the EO.

The cross-species analysis has revealed that the EO transcriptome is more variable in terms of gene expression levels across species than the SM transcriptome. The functional annotation indicates that the most diverging functional classes across species in the EO include “metabolism of fatty acids” and “ion transport”.

In order to better understand the role played by the differentially expressed gene identified in this study, the onset of transgenic experiments (e.g., knockdown) will be necessary either in fully established model organisms (*D. rerio*), or in emerging models for electric fish (*E. electricus*).

## Methods

### Specimen collection

Several specimens of *C. compressirostris*, *C. tshokwe* and *G. petersii* were collected in the wild during a sampling campaign conducted at the Congo River rapids south of Brazzaville (Republic of the Congo, August/September 2012). For the present study, adult female specimens for each species were selected, kept in captivity for a maximum period of 2 weeks and then euthanized for tissue sample collection. Gender and sexual maturity were assessed after dissection by checking for the presence of mature ovaries in the selected specimens. Electric organ and skeletal muscle tissue samples were dissected from the caudal peduncle and the posterior trunk musculature respectively and immediately transferred into RNAlater® (Life Technologies). The research followed internationally recognized guidelines and applicable national legislation. We received ethical approval from the deputy of animal welfare of the University of Potsdam.

### RNA extraction and cDNA library preparation

The dissected tissues were processed at the University of Potsdam for RNA extraction: they were first removed from the RNAlater®-containing vials; shock frozen in liquid nitrogen and then homogenized into a buffer containing guanidine isothiocyanate and β-mercaptoethanol using a Mini-beadbeater-1 (Biospec). Total RNA was extracted using the RNeasy® Mini Kit (Qiagen), RNA quality and concentration was inspected using a Fragment Analyzer™ (Advanced Analytical Technologies, Inc.).

For the present study eight cDNA libraries were selected for sequencing (one library per species per tissue; two biological replicates), each library was obtained by pooling the total RNA from a minimum of two to a maximum of 4 different individuals (see Additional file 1). The paired-end (100 nt), strand-specific cDNA libraries were prepared using the NEXTflex™ Directional RNA-Seq Kit V2 (dUTP based) (Bioo Scientific); preparation was performed in six steps: i) mRNA enrichment from total RNA via polyA selection; ii) fragmentation; iii) first and second strand syntheses; iv) A-tailing; v) adapter and barcode ligation and vi) PCR amplification. Fragment size distribution and quality was estimated using an Agilent 2100 Bioanalyzer with the High Sensitivity DNA Chip.

### Transcriptome sequencing, assembly and annotation

Transcriptome sequencing was performed at the Max Delbrück Center for Molecular Medicine; the multiplexed cDNA libraries were sequenced using one lane of an Illumina HiSeq2000 sequencing system. After sequencing, the resulting raw reads were subject to five processing steps using the program Flexbar v2.4 [37] : i) filtering reads with uncalled bases; ii) trimming of reads at 3′-end to get a minimum average Phred quality score of 20; iii) barcode detection, removal and reads separation; iv) adapter detection and removal, and v) filtering of reads shorter than 20 bp after trimming. Quality control of both raw and processed reads was performed with FastQC v0.10.1 (Babraham Bioinformatics).

**Fig. 9:**
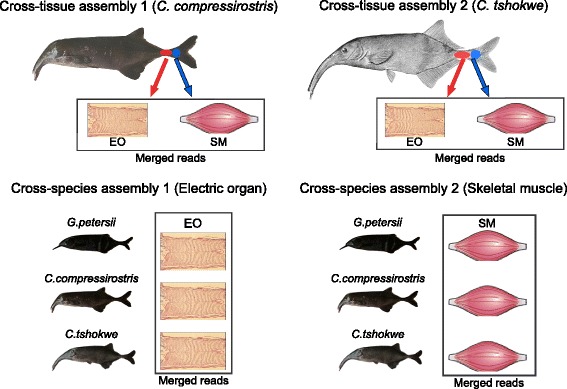
Assembly schemes for the cross-tissue and cross-species comparison

The processed reads were assembled *de novo* (i.e., without using a reference genome) with Trinity r20131110 [38] (kmer length = 25). Two reference transcriptomes were produced from *C. compressirostris* and *C. tshokwe* respectively, by assembling together the reads obtained from the EO and SM libraries. Combining all reads across all tissues and all biological replicates for each species (cross-tissue assembly), or across all species and all replicates for each tissue (cross-species assembly) (Fig. 9) into a single RNA-seq dataset, allows to correctly compare transcript abundances from the analysed tissues or species by aligning the short reads from each library independently onto the same set of reference transcripts (see below and Additional files 2, 3, 4, 5, 6 and 7 for more details) [39].

Transcriptome annotation was conducted using the stand-alone version of the blastx algorithm implemented in Blast + v2.2.29 [40] (E-value cutoff = 10^−10^) against the proteome of *Danio rerio* (Uniprot ID = UP000000437). Likely coding sequences were extracted from Trinity transcripts using TransDecoder (http://transdecoder.github.io/) and the longest translated Open Reading Frames (ORFs) were reported (Table 1). Protein domains were searched on the PFAM database (Pfam-A.hmm available at http://pfam.xfam.org/) using HMMER v3.1b1. The retrieved ORFs were later annotated by “blasting” them against the SwissProt (http://web.expasy.org/docs/swiss-prot_guideline.html) database using the blastp algorithm. Transcripts’ completeness was assessed by computing the proportion of transcripts and ORFs that matched to full-length top hits in their respective searches using the Perl script “analyze_blastPlus_topHit_coverage.pl” (provided with Trinity) (Fig. 2 and Additional file 8) [39].

The use of a single reference species for annotation is sub-optimal in terms of number of retrieved orthologs if compared to other methods like iterative BLAST searches to multiple species [41], however the use of a long-time established model organism (*D. rerio*) facilitates the enrichment analysis and the identification of experimental evidence for the functional role of a given gene.

### Transcript abundance quantification and DE-analysis

Short reads were individually mapped to their respective transcriptome assemblies using Bowtie v1.0.0 [42] with default parameters. Gene expression levels were estimated using RSEM v1.2.12 [43]. Putative transcript artifacts and lowly expressed transcripts were filtered out using the Perl script “filter_fasta_by_rsem_values.pl” (provided with Trinity).

Principal component analyses of cross-species data was performed on a matrix of expression values of 14,436 genes with non-zero values in both assemblies, using the function “prcomp” in R. The same dataset was used for building a distance matrix of Spearman’s correlation coefficients (ρ) which was then subjected to neighbour-joining tree construction with the function “nj” in R (bootstrap = 10,000). Differential expression analysis was performed using the Bioconductor package edgeR [44] (minimum fold change = 4, *p*-value cutoff = 0.001 after FDR correction). The differentially expressed transcripts were then subject to an enrichment analysis using the Cytoscape plugin ClueGO v2.1.4 [45, 46], in order to identify over-represented functional categories from the Gene Ontology (GO) database [47] (http://geneontology.org), as well as over-represented biological pathways from KEGG (http://www.genome.jp/kegg/) and Reactome (http://www.reactome.org/) [48, 49]. Statistical significance was assessed using a Fisher’s exact test with FDR *p*-value correction (≤0.05). Cross-species data were partitioned into expression clusters using a k-means algorithm (*k* = 4), implemented in a perl script provided with Trinity.

## Data availability

All the Illumina reads used for this study are available at the Sequence Read Archive (SRA; http://www.ncbi.nlm.nih.gov/sra), under the accession number SRP050174.

## Additional files

Additional file 1:
**Sequencing statistics.** For each of the eight produced libraries we report: the number of pooled individuals, the number of pre- and post- processing read pairs, and the percent of retained reads after quality filtering. (DOCX 17 kb) Additional file 2:
**Blast results of** ***C. compressirostris*** **transcriptome assembly.** Tabular blast report showing the results of the blastx comparison between the transcriptome of *C. compressirostris* and the proteome of *D. rerio*. (TXT 14648 kb) Additional file 3:
**Blast results of** ***C. tshokwe*** **transcriptome assembly.** Tabular blast report showing the results of the blastx comparison between the transcriptome of *C. tshokwe* and the proteome of *D. rerio*. (TXT 19887 kb) Additional file 4:
**Changes in gene expression between EO and SM for** ***C. compressirostris*** **transcriptome.** Results of the edgeR DE-analysis. The table reports the fold-change in the expression levels and relative uncorrected and corrected *p*-values between EO and SM for all genes in the *C. compressirostris* transcriptome. (TXT 5878 kb) Additional file 5:
**Changes in gene expression between EO and SM for** ***C. tshokwe*** **transcriptome.** Results of the edgeR DE-analysis. The table reports the fold-change in the expression levels and relative uncorrected and corrected p-values between EO and SM for all genes in the *C. tshokwe* transcriptome. (TXT 7336 kb) Additional file 6:
**Transcripts’ length distribution of the** ***C. compressirostris*** **Trinity assembly.** (PDF 24 kb) Additional file 7:
**Transcripts’ length distribution of the** ***C. tshokwe*** **Trinity assembly.** (PDF 5 kb) Additional file 8:
**Distribution of length coverage between retrieved ORFs and reference database (SwissProt).** (PDF 44 kb) Additional file 9:
**List of differentially expressed genes belonging to the class “signal transduction”, obtained from the cross-tissue comparison.** (DOCX 39 kb)
