# Supplementary material for: Cross-tissue and cross-species analysis of gene expression in skeletal muscle and electric organ of African weakly-electric fish (Teleostei; Mormyridae)
Source: BMC Genomics. 2015 Sep 3;16(1):668. doi: 10.1186/s12864-015-1858-9 (PMC4558960; doi:10.1186/s12864-015-1858-9)
Supplement: Additional file 1: — Sequencing statistics. For each of the eight produced libraries we report: the number of pooled individuals, the number of pre- and post- processing read pairs, and the percent of retained reads after quality filtering. (DOCX 17 kb) [file 12864_2015_1858_MOESM1_ESM.docx]

| Library name | # of pooled individuals | # of raw reads  per pair | # of processed reads  per pair | % of retained  reads |
| --- | --- | --- | --- | --- |
| C_comp_EO_rep1 | 2 | 19,999,194 | 17,847,585 | 89.2 |
| C_comp_EO_rep2 | 3 | 18,303,901 | 15,645,817 | 85.5 |
| C_comp_MU_rep1 | 2 | 42,123,838 | 37,256,680 | 88.4 |
| C_comp_MU_rep2 | 3 | 26,763,905 | 23,932,197 | 89.4 |
| C_tsho_EO_rep1 | 3 | 24,225,505 | 21,703,048 | 89.6 |
| C_tsho_EO_rep2 | 4 | 41,947,351 | 37,294,087 | 88.9 |
| C_tsho_MU_rep1 | 2 | 20,963,023 | 18,758,922 | 89.5 |
| C_tsho_MU_rep2 | 4 | 72,726,155 | 64,419,314 | 88.6 |
| G_pet_EO_rep1 | 3 | 21,224,019 | 18,910,601 | 89.1 |
| G_pet_EO_rep2 | 3 | 34,232,579 | 30,638,159 | 89.5 |
| G_pet_MU_rep1 | 3 | 25,753,014 | 22,868,677 | 88.8 |
| G_pet_MU_rep2 | 3 | 22,780,873 | 20,320,459 | 89.2 |

Supplemental table 1: information for the sequenced libraries.

C_comp = *C. compressirostris*; C_tsho = *C. tshokwe*; EO = Electric Organ; MU = Skeletal Muscle; rep* = replicate number.
