# Supplementary figures and images for: Cross-tissue and cross-species analysis of gene expression in skeletal muscle and electric organ of African weakly-electric fish (Teleostei; Mormyridae)
Source: BMC Genomics. 2015 Sep 3;16(1):668. doi: 10.1186/s12864-015-1858-9 (PMC4558960; doi:10.1186/s12864-015-1858-9)

# Contig length distribution: *C. compressirostris*

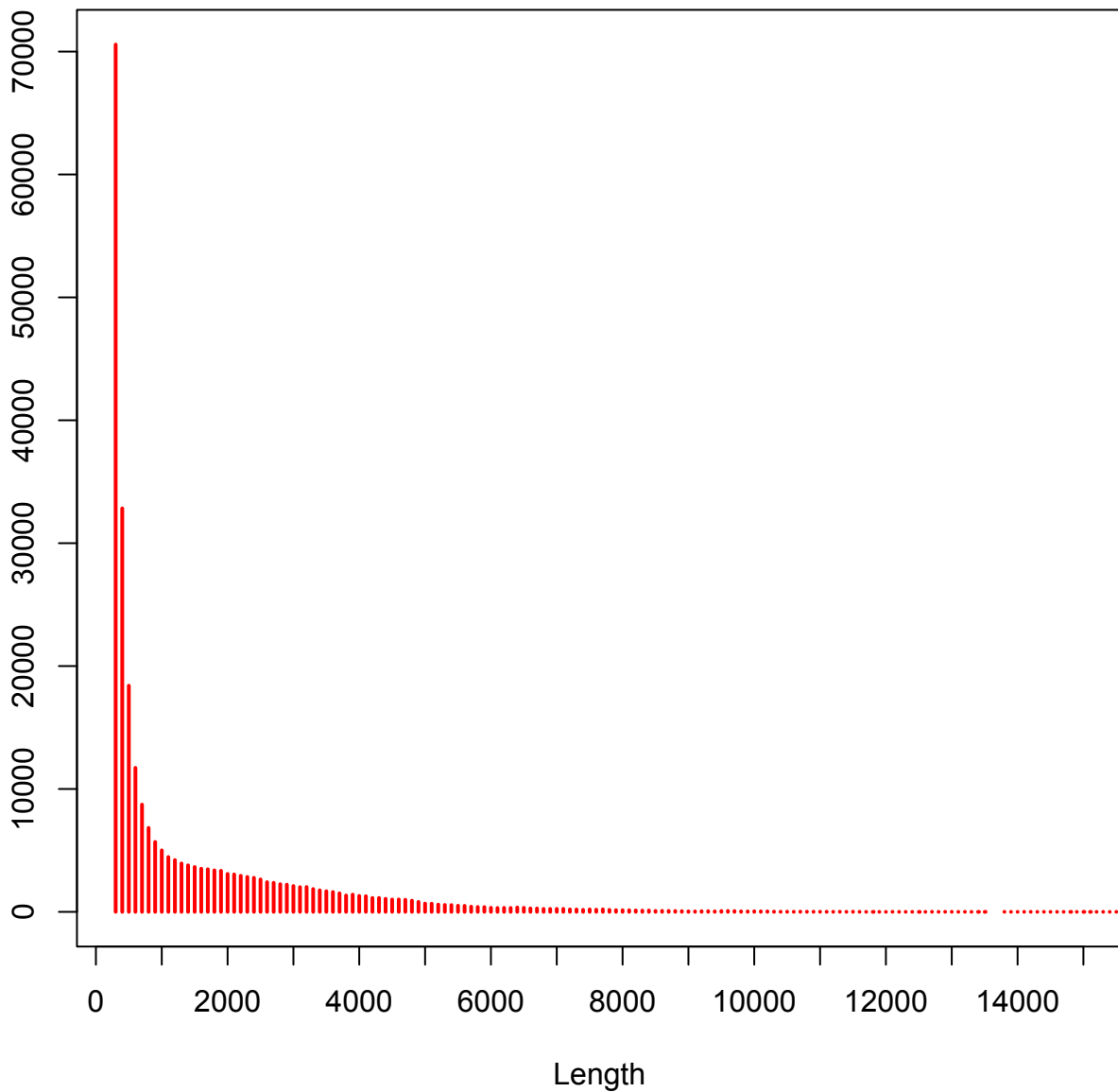

Supplement: Additional file 6: — Transcripts’ length distribution of the C. compressirostris Trinity assembly. (PDF 24 kb) [file 12864_2015_1858_MOESM6_ESM.pdf]

**Contig length distribution: *C. tshokwe***

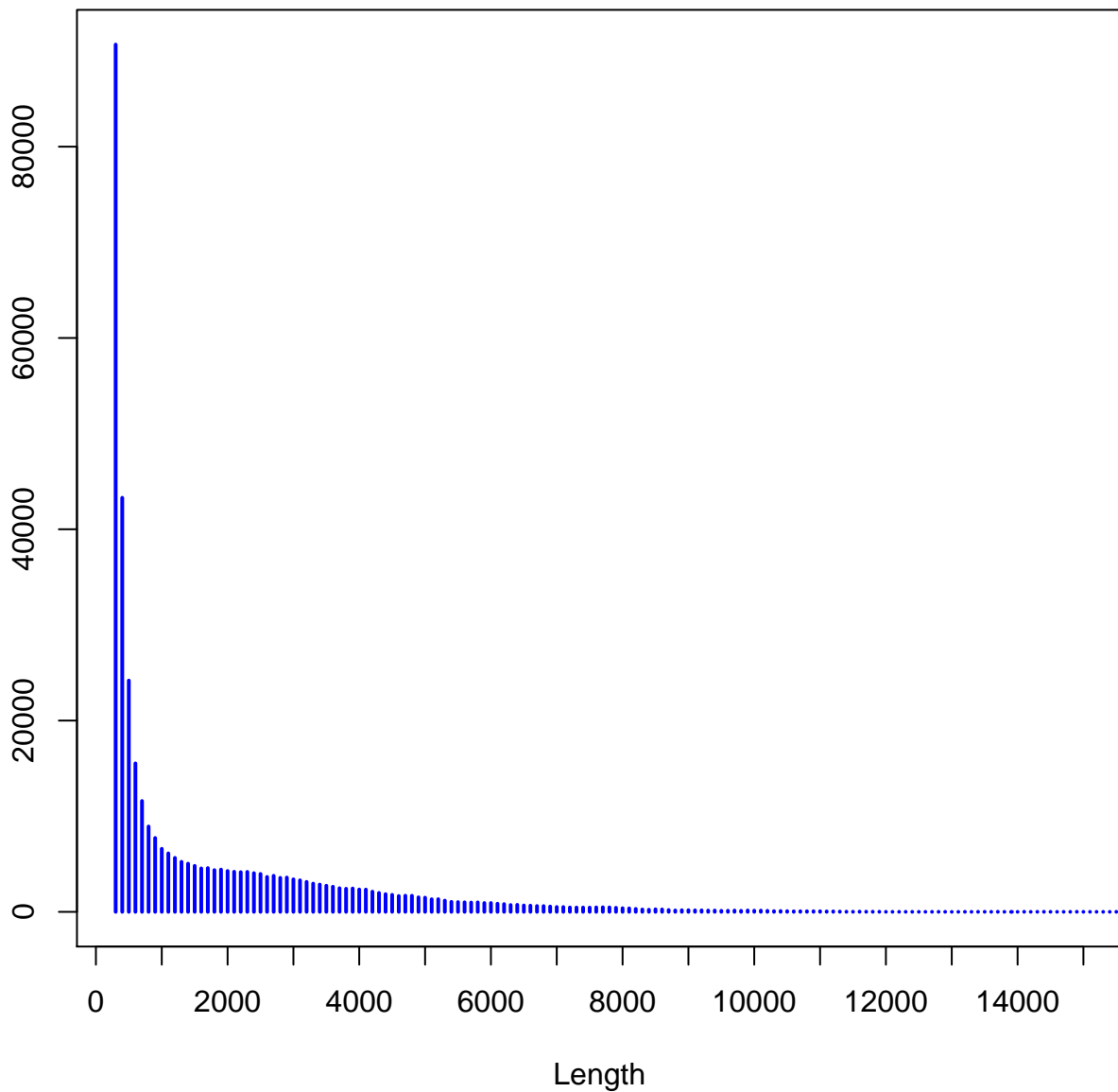

Supplement: Additional file 7: — Transcripts’ length distribution of the C. tshokwe Trinity assembly. (PDF 5 kb) [file 12864_2015_1858_MOESM7_ESM.pdf]

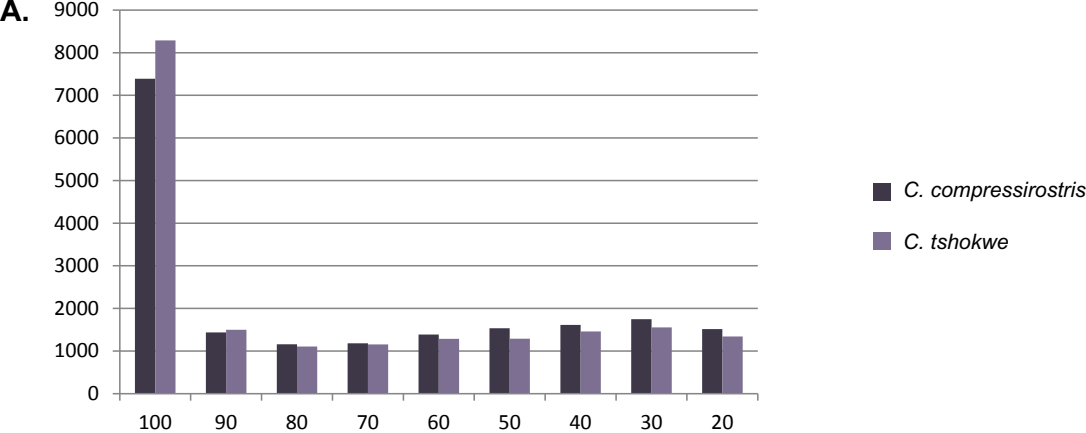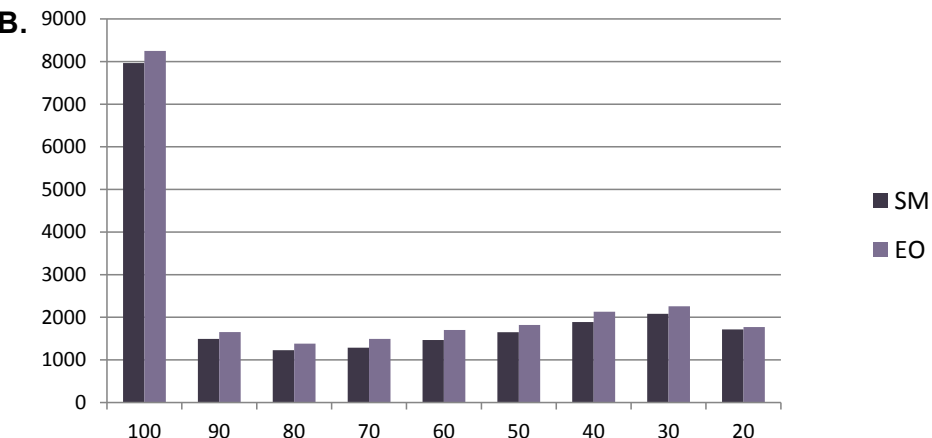

Supplement: Additional file 8: — Distribution of length coverage between retrieved ORFs and reference database (SwissProt). (PDF 44 kb) [file 12864_2015_1858_MOESM8_ESM.pdf]
