# Supplementary material for: Cross-tissue and cross-species analysis of gene expression in skeletal muscle and electric organ of African weakly-electric fish (Teleostei; Mormyridae)
Source: BMC Genomics. 2015 Sep 3;16(1):668. doi: 10.1186/s12864-015-1858-9 (PMC4558960; doi:10.1186/s12864-015-1858-9)
Supplement: Additional file 9: — List of differentially expressed genes belonging to the class “signal transduction”, obtained from the cross-tissue comparison. (DOCX 39 kb) [file 12864_2015_1858_MOESM9_ESM.docx]

**Signal transduction**

For each of the shared differentially expressed gene are reported: the gene and protein names obtained from the top hit blast results against the proteome of *D. rerio*; whether it is up(+)- or down(-)- regulated in the EO; its function or pathway (or both when available); the phenotypic effect on *D. rerio* of its mis-expression (when available).

| Gene | Protein Name | Expression in EO | Pathway | Disrupted Phenotype | Reference |
| --- | --- | --- | --- | --- | --- |
| arhgef7a | Rho guanine nucleotide exchange factor (GEF) 7a | + | Regulation of actin cytoskeleton; Signaling by GPCR; Signaling by FGFR |  |  |
| arhgef7b | Rho guanine nucleotide exchange factor (GEF) 7b | + | Regulation of actin cytoskeleton; Signaling by GPCR; Signaling by FGFR |  |  |
| catip | ciliogenesis associated TTC17 interacting protein | + | actin filament polymerization | Imperfect ciliogenesis | [1] |
| fgf8a | fibroblast growth factor 8a | + | Signaling by FGFR; Regulation of actin cytoskeleton | Imperfect morphogenesis | [2] |
| kal1b | Kallmann syndrome 1b sequence | + | Signaling by FGFR |  |  |
| gpr22 | G protein-coupled receptor 22 | + | G-protein coupled receptor signaling pathway |  |  |
| reps2 | RALBP1 associated Eps domain containing 2 | + | EGFR1 Signaling Pathway |  |  |
| olfcs2 | olfactory receptor C family, s2 | + | G-protein coupled receptor signaling pathway |  |  |
| olfm2a | olfactomedin 2a | + | neural crest cell development | Central nervous system development | [3] |
| opn3 | Opsin 3 | + | G-protein coupled receptor signaling pathway |  |  |
| pcsk5b | proprotein convertase subtilisin/kexin type 5b | + | Signaling by FGFR; Signaling by GPCR; NGF processing |  |  |
| pik3cg | phosphatidylinositol-4,5-bisphosphate 3-kinase, catalytic subunit gamma | + | Toll-like receptor signaling pathway |  |  |
| prkar1b | protein kinase, cAMP-dependent, regulatory, type I, beta | + | G-protein coupled receptor signaling pathway |  |  |
| rgs11 | regulator of G-protein signaling 11 | + | G-protein coupled receptor signaling pathway |  |  |
| arhgap44 | Rho GTPase activating protein 44 | + | G-protein coupled receptor signaling pathway |  |  |
| rapgefl1 | Rap guanine nucleotide exchange factor (GEF)-like 1 | + | G-protein coupled receptor signaling pathway |  |  |
| gab3 | GRB2-Associated Binding Protein 3 | + | Akt Signaling Pathway |  |  |
| tpbga | trophoblast glycoprotein a | + | negative regulation of canonical Wnt signaling pathway |  |  |
| trabd2b | TraB domain containing 2B | + | Wnt signaling pathway |  |  |
| twf1b | twinfilin actin-binding protein 1b | + | negative regulation of actin filament polymerization |  |  |
| wwc1 | WW and C2 domain containing 1 | + | G-protein coupled receptor signaling pathway |  |  |
| cdk14 | cyclin-dependent kinase 14 | + | Transcriptional misregulation in cancer |  |  |
| angpt1 | angiopoietin 1 | - | ERK Signaling; Akt Signaling; TGF-Beta Pathway; Hedgehog signaling | Imperfect angiogenesis | [4] |
| asb10 | ankyrin repeat and SOCS box containing 10 | - | Class I MHC mediated antigen processing and presentation |  |  |
| calcoco1 | calcium binding and coiled-coil domain 1 | - | Wnt signaling pathway |  |  |
| ccng1 | cyclin G1 | - | p53 signaling pathway; G-protein coupled receptor signaling pathway |  |  |
| dapk2a | death-associated protein kinase 2a | - | Regulation of Apoptosis |  |  |
| dusp22b | dual specificity phosphatase 22b | - | TGF-Beta Pathway |  |  |
| fhl1a | four and a half LIM domains 1a | - | Delta-Notch Signaling Pathway | Abnormal cardiac function | [5] |
| flncb | filamin C, gamma b (actin binding protein 280) | - | MAPK signaling pathway | Myofibril disruption | [6] |
| homer1b | homer homolog 1b (Drosophila) | - | FoxO signaling pathway; Regulation of calcium homeostasis | defective phenotypes in fast muscle | [7] |
| igf1 | insulin-like growth factor 1 | - | Development IGF 1 receptor signaling; G-protein coupled receptor signaling pathway |  |  |
| il13ra2 | interleukin 13 receptor, alpha 2 | - | Akt Signaling; TGF-Beta Pathway; ERK Signaling |  |  |
| klhl41b | kelch-like family member 41b | - | Regulation of myoblast differentiation | myofibrillar disorganization | [8] |
| lnx1 | ligand of numb-protein X 1 | - | Notch signaling pathway |  |  |
| lypd6 | LY6/PLAUR domain containing 6 | - | positive regulation of canonical Wnt signaling pathway | caudal fin decreased size; trunk decreased size | [9] |
| myoc | myocilin | - | Wnt signaling pathway |  |  |
| ndp | Norrie disease (pseudoglioma) | - | Wnt signaling pathway |  |  |
| pde7a | phosphodiesterase 7A | - | G-protein coupled receptor signaling pathway |  |  |
| pmp22a | peripheral myelin protein 22a | - | Neural Crest Differentiation |  |  |
| ppdpfa | pancreatic progenitor cell differentiation and proliferation factor a | - | Negative regulation of RA signaling pathway | Abnormal pancreas development | [10] |
| prkg1b | protein kinase, cGMP-dependent, type Ib | - | beta-catenin independent WNT signaling |  |  |
| sbk3 | SH3 domain binding kinase family, member 3 | - | MAPK signaling pathway |  |  |
| mras | muscle RAS oncogene homolog | - | MAPK signaling pathway; G-protein coupled receptor signaling pathway |  |  |
| plekha1 | pleckstrin homology domain containing, family A (phosphoinositide binding specific) member 1 | - | Class I PI3K signaling events |  |  |
| spon2a | spondin 2a, extracellular matrix protein | - | Integrin Pathway; ERK Signaling |  |  |
| tacr1a | tachykinin receptor 1a | - | G-protein coupled receptor signaling pathway |  |  |
| txlnba | taxilin beta a | - | TNF-alpha/NF-kB Signaling Pathway |  |  |
| txlnbb | taxilin beta b | - | TNF-alpha/NF-kB Signaling Pathway |  |  |
| ywhag1 | 3-monooxygenase/tryptophan 5-monooxygenase activation protein, gamma polypeptide 1 | - | Cell cycle | Reduced brain size; Increased heart tube diameter | [11] |

**References:**

1. Bontems F, Fish RJ, Borlat I, Lembo F, Chocu S, Chalmel F, Borg J-P, Pineau C, Neerman-Arbez M, Bairoch A, Lane L: **C2orf62 and TTC17 are involved in actin organization and ciliogenesis in zebrafish and human.** *PLoS One* 2014, **9**:e86476.
2. Albertson RC, Yelick PC: **Fgf8 haploinsufficiency results in distinct craniofacial defects in adult zebrafish.** *Dev Biol* 2007, **306**:505–15.
3. Lee J-A, Anholt RRH, Cole GJ: **Olfactomedin-2 mediates development of the anterior central nervous system and head structures in zebrafish.** *Mech Dev* 2008, **125**:167–81.
4. Lamont RE, Vu W, Carter AD, Serluca FC, MacRae CA, Childs SJ: **Hedgehog signaling via angiopoietin1 is required for developmental vascular stability.** *Mech Dev* 2010, **127**:159–68.
5. Xie H, Fan X, Tang X, Wan Y: **The LIM Protein fhlA is Essential for Heart Chamber Development in Zebrafish Embryos**. *Curr Mol Med* 2013, **13**:979–992.
6. Ruparelia A a, Zhao M, Currie PD, Bryson-Richardson RJ: **Characterization and investigation of zebrafish models of filamin-related myofibrillar myopathy.** *Hum Mol Genet* 2012, **21**:4073–83.
7. Lin C-Y, Chen J-S, Loo M-R, Hsiao C-C, Chang W-Y, Tsai H-J: **MicroRNA-3906 regulates fast muscle differentiation through modulating the target gene homer-1b in zebrafish embryos.** *PLoS One* 2013, **8**:e70187.
8. Gupta VA, Ravenscroft G, Shaheen R, Todd EJ, Swanson LC, Shiina M, Ogata K, Hsu C, Clarke NF, Darras BT, Farrar MA, Hashem A, Manton ND, Muntoni F, North KN, Sandaradura SA, Nishino I, Hayashi YK, Sewry CA, Thompson EM, Yau KS, Brownstein CA, Yu TW, Allcock RJN, Davis MR, Wallgren-Pettersson C, Matsumoto N, Alkuraya FS, Laing NG, Beggs AH: **Identification of KLHL41 Mutations Implicates BTB-Kelch-Mediated Ubiquitination as an Alternate Pathway to Myofibrillar Disruption in Nemaline Myopathy.** *Am J Hum Genet* 2013, **93**:1108–17.
9. Özhan G, Sezgin E, Wehner D, Pfister AS, Kühl SJ, Kagermeier-Schenk B, Kühl M, Schwille P, Weidinger G: **Lypd6 enhances Wnt/β-catenin signaling by promoting Lrp6 phosphorylation in raft plasma membrane domains.** *Dev Cell* 2013, **26**:331–45.
10. Jiang Z, Song J, Qi F, Xiao A, An X, Liu N, Zhu Z, Zhang B, Lin S: **Exdpf is a key regulator of exocrine pancreas development controlled by retinoic acid and ptf1a in zebrafish.** *PLoS Biol* 2008, **6**:e293.
11. Komoike Y, Fujii K, Nishimura A, Hiraki Y, Hayashidani M, Shimojima K, Nishizawa T, Higashi K, Yasukawa K, Saitsu H, Miyake N, Mizuguchi T, Matsumoto N, Osawa M, Kohno Y, Higashinakagawa T, Yamamoto T: **Zebrafish gene knockdowns imply roles for human YWHAG in infantile spasms and cardiomegaly.** *Genesis* 2010, **48**:979–992.
